# Evaluating bone biopsy quality by technique in an animal model

**DOI:** 10.1016/j.redii.2022.100008

**Published:** 2022-05-21

**Authors:** Corey K Ho, David Gimarc, Hsieng-Feng Carroll, Michael Clay, Jeffrey Schowinsky, MK Jesse, Amanda M Crawford, Carrie B Marshall

**Affiliations:** a University of Colorado – Anschutz Medical Campus, Department of Radiology, 12401 E 17th Ave, Aurora, CO 80045, USA; b University of Colorado – Anschutz Medical Campus, Department of Pathology, 12401 E 17th Ave, Aurora, CO 80045, USA; c University of Utah – Department of Radiology and Imaging Sciences, University of Utah Hospital, 50 2030 E, Salt Lake City, UT 84132, USA

**Keywords:** Bone, Biopsy, Artifact

## Abstract

**Rationale and Objectives:**

Powered bone biopsy technique is popular due to its ease of use. However, there is conflicting evidence regarding the diagnostic quality of the samples. The purpose of this study is to evaluate the diagnostic adequacy of different bone biopsy devices and techniques as it relates to the frequency of sample artifacts.

**Materials and Methods:**

Bone biopsy was performed on same-day processed lamb femora using the following techniques: manual, pulsed powered and full powered. Ten samples were collected using each method by a single musculoskeletal-trained radiologist and were reviewed by 3 blinded pathologists. Samples were compared across multiple categories: length, bone dust, thermal/crush artifact, cellular morphology, fragmentation, and diagnostic acceptability. Bayesian Multilevel Nonlinear Regression models were performed assessing the association between the techniques across the categories.

**Results:**

Statistical analysis revealed that the manual technique outperformed any powered technique across all categories: decreased thermal/crush artifact (*P* = 0.014), decreased bone dust (p<0.001), better cellular morphology (*P* = 0.005), less fragmentation (*P* < 0.0001) and better diagnostic acceptability (*P* < 0.0001).

**Conclusion:**

Manually obtained bone biopsy samples generally produce a more diagnostic sample as compared to powered techniques in an animal model. Given these results, manual bone biopsy methods should be encouraged after consideration for lesion composition, difficulty of access and the patient's overall condition.

## 1 Introduction

Image-guided percutaneous core needle biopsy is a crucial first step in the diagnostic work up of many osseous lesions [1]. A variety of commercially available tools exist for the acquisition of core samples. There are conflicting opinions and reports regarding the quality of bone specimens obtained by manual versus powered biopsy devices.

Accuracy of pathologic analysis of bone specimens can be impacted by artifacts and alterations incurred during the biopsy procedure. While some studies suggest that a rotary-powered device yields a cohesive sample with rare exceptions [2], [3], [4], other studies caution that drill-assisted devices can contribute to crush artifact and shorter lengths of assessable marrow [5], [6], [7], [8]. Operators of the powered system have voiced anecdotal concerns over thermal artifact or burn related to friction, however several studies assessing specimen artifacts did not identify thermal damage [5,9,10]. Prior studies have evaluated sample adequacy between powered versus manual techniques [3,7,[11], [12], [13]], with differing results. Currently, comparison between bone biopsy using manual and varied powered techniques in an animal model has not been performed.

The purpose of this study is to evaluate the diagnostic adequacy of different bone biopsy devices and techniques as it relates to the frequency of sample artifacts utilizing an animal model.

## 2 Materials and methods

### 2.1 Technique

To compare diagnostic quality and the incidence of artifacts during bone biopsy, an animal model was used. Same-day processed eviscerated lamb femora were used for this study to preserve cell viability and for its well-documented similarity to human bone [14], [15], [16]. As this study utilized byproducts from a food processing plant destined for refuse, it is not considered as using animal subjects, and as such, IRB approval was not necessary. A powered coaxial bone biopsy system, Arrow® OnControl® 10-gauge access & 12-gauge biopsy set, (Teleflex®, Morrisville, NC) as well as a manual coaxial biopsy system, Bonopty® 12-gauge access & 13-gauge biopsy set (Apriomed®, Derry, NH) were used. Two techniques were utilized to determine if speed was a factor with the powered device: pulsed powered access & full powered access. This modification was used as the powered system does not have a controllable throttle. When using the pulsed powered technique, the trigger was turned on and immediately released when rotation was achieved. This was repeated until targeted depth was achieved.

**Figure 1:**
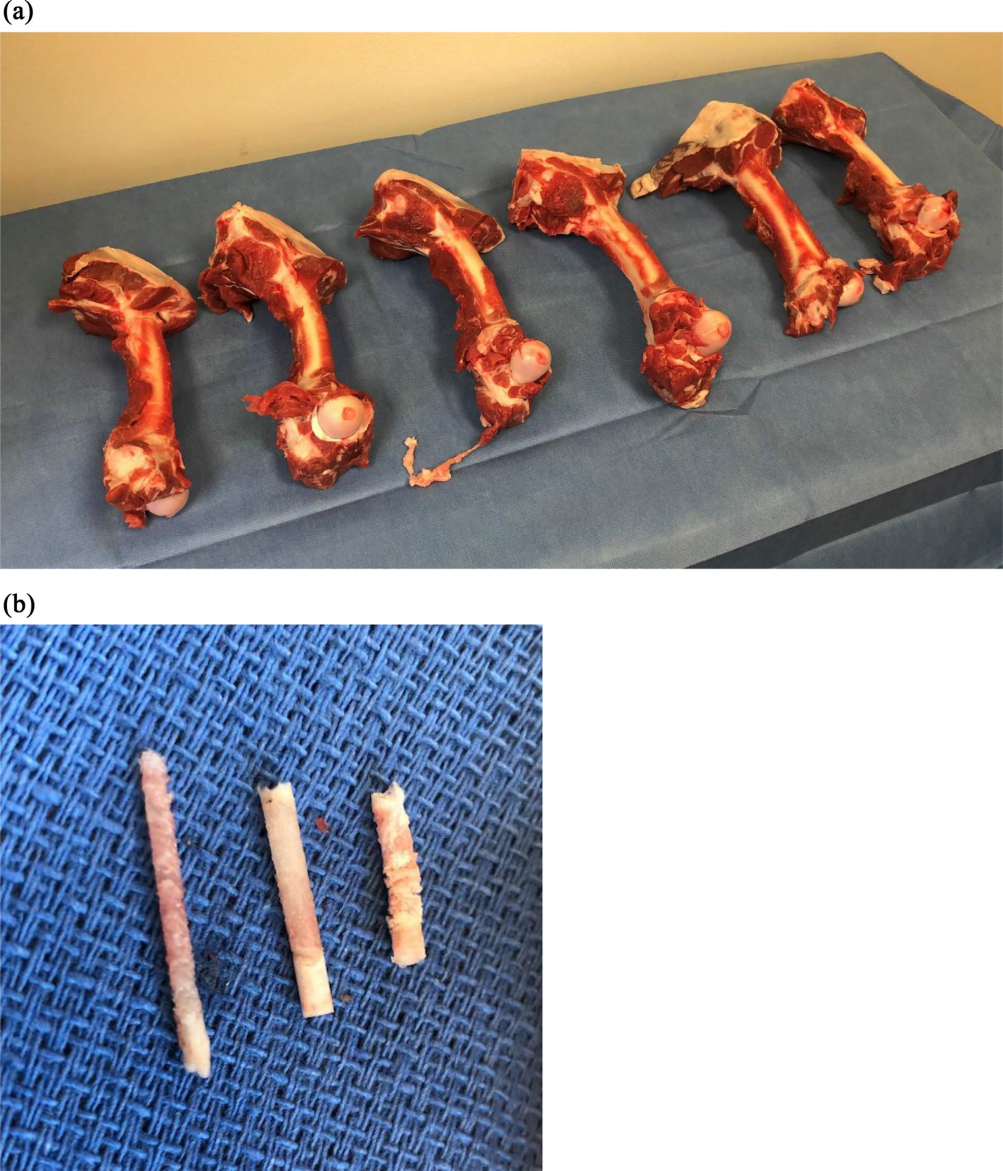
Biopsy specimens. (a) Examples of the same-day processed lamb femora used for sampling. (b) Examples of tissue obtained using different tools. On the left, is an example of the manual technique. The middle sample was obtained with a full-powered technique. On the right, is an example of the pulsed-powered technique.

Initial access through cortical bone was obtained with power tool assistance for the powered system and with a mallet for the manual system. Then biopsy devices were placed coaxially within their respective system. For the pulsed and full powered techniques, a sample depth of 2 cm was targeted to obtain and retrieve the samples. For the manual technique, a sample depth of 2 cm was also targeted using a mallet to obtain the sample. This process was repeated ten times for each technique at the femoral condyles. The bones were obtained the same day of slaughter to limit any post-mortem changes. Each femoral condyle was accessed four times in adjacent locations without overlapping tracts. The samples were expelled from the needle per manufacturer instructions. For the powered system the appropriate pusher was placed anterograde for the pulsed and full powered techniques. For the manual system, the pusher system was placed retrograde. A single musculoskeletal fellowship-trained radiologist with 4 years of experience obtained all the samples. Images of source tissue and representative specimens are depicted in Figure 1.

### 2.2 Pathologic analysis

Immediately following retrieval, the samples were placed directly into 50 mL of 10% neutral buffered formalin in randomly numbered containers. The cores were formalin-fixed for six days and subsequently grossly examined and measured in mm (longest piece if fragmented) by a single pathologist with 10 years of experience. The samples were submerged in Thermo Scientific Richard-Allan Scientific Decalcifying Solution (EDTA and dilute HCl) for 30-40 minutes or until pliable. They were then rinsed in tap water and returned to formalin for later tissue processing on a Tissue Tek VIP processor using a standard protocol (10.25 hours total processing time). These were paraffin embedded and cut onto glass slides, subsequently stained with hematoxylin and eosin.

**Figure 2:**
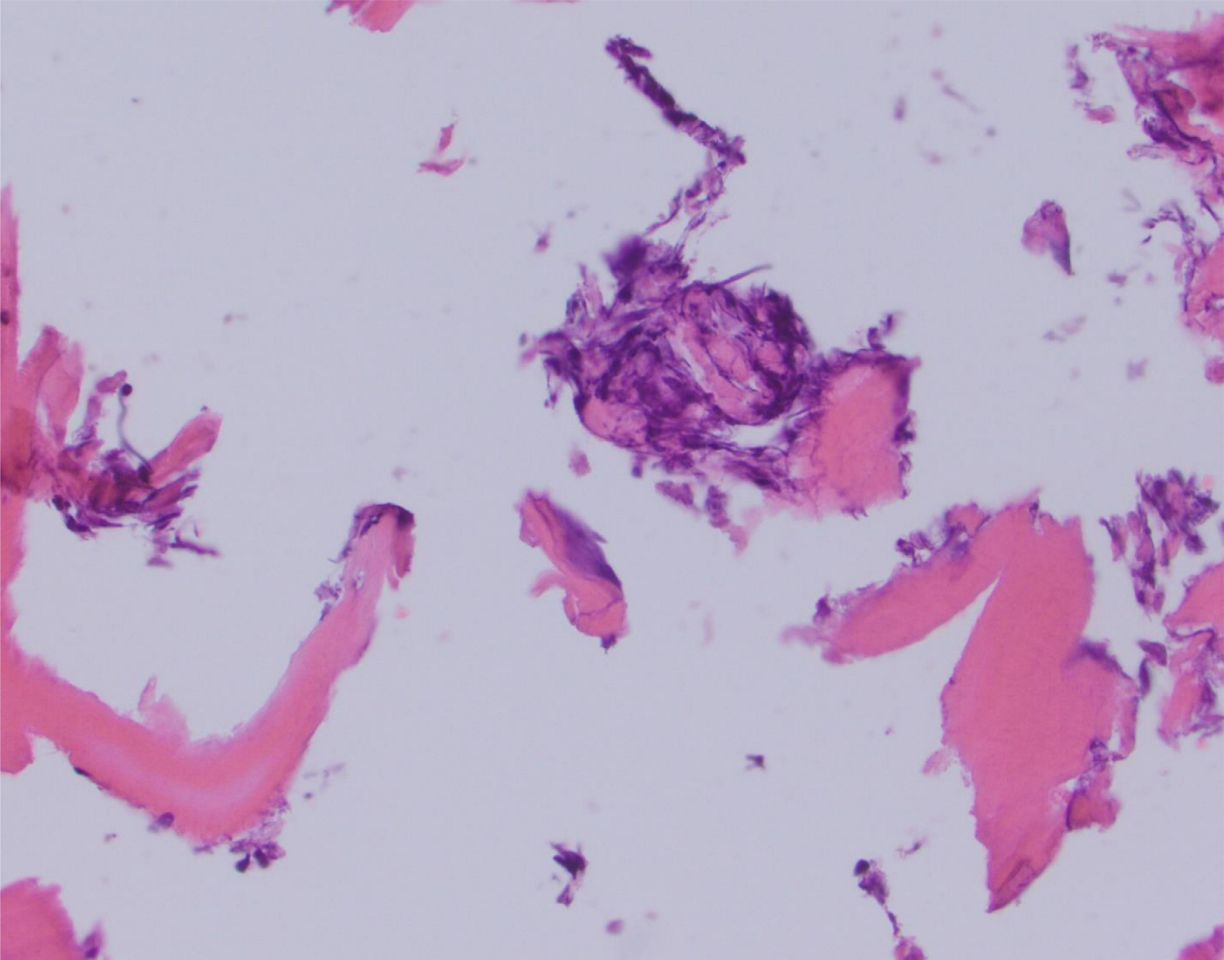
Crush Artifact. Hematoxylin and eosin-stained slide at high power depicts an example of crush artifact (near the center of the slide) with compaction of the cellular material. This was obtained with the manual technique.

**Figure 3:**
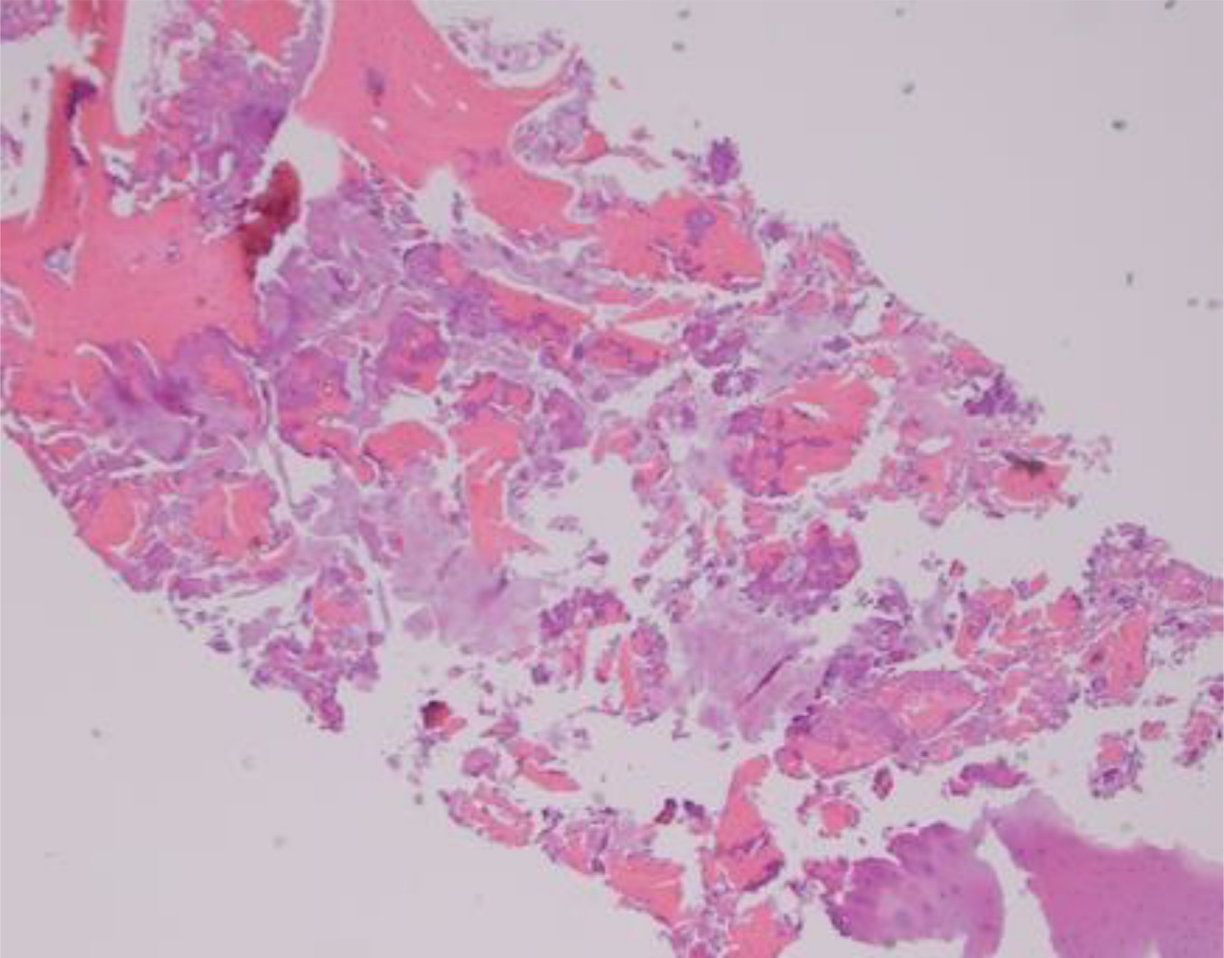
Bone dust. Hematoxylin and eosin-stained slide at high power depicts an example of bone dust with microscopic fragments of pulverized bone associated with instrumentation. This was obtained with the pulsed-powered technique.

**Figure 4:**
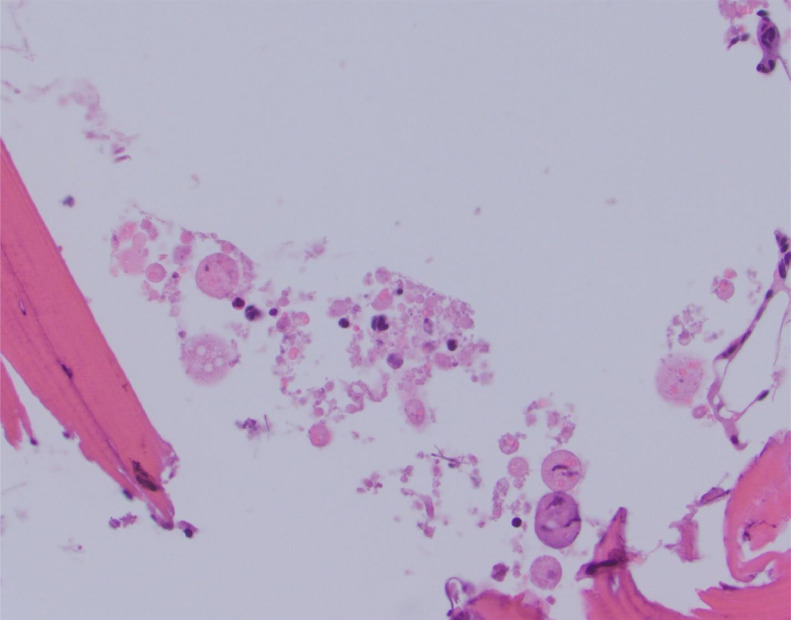
Cellular morphology. Hematoxylin and eosin-stained slide at high power depicts poor cellular morphology with altered shapes of cells and anatomy. This was obtained with the pulsed-powered technique.

**Figure 5:**
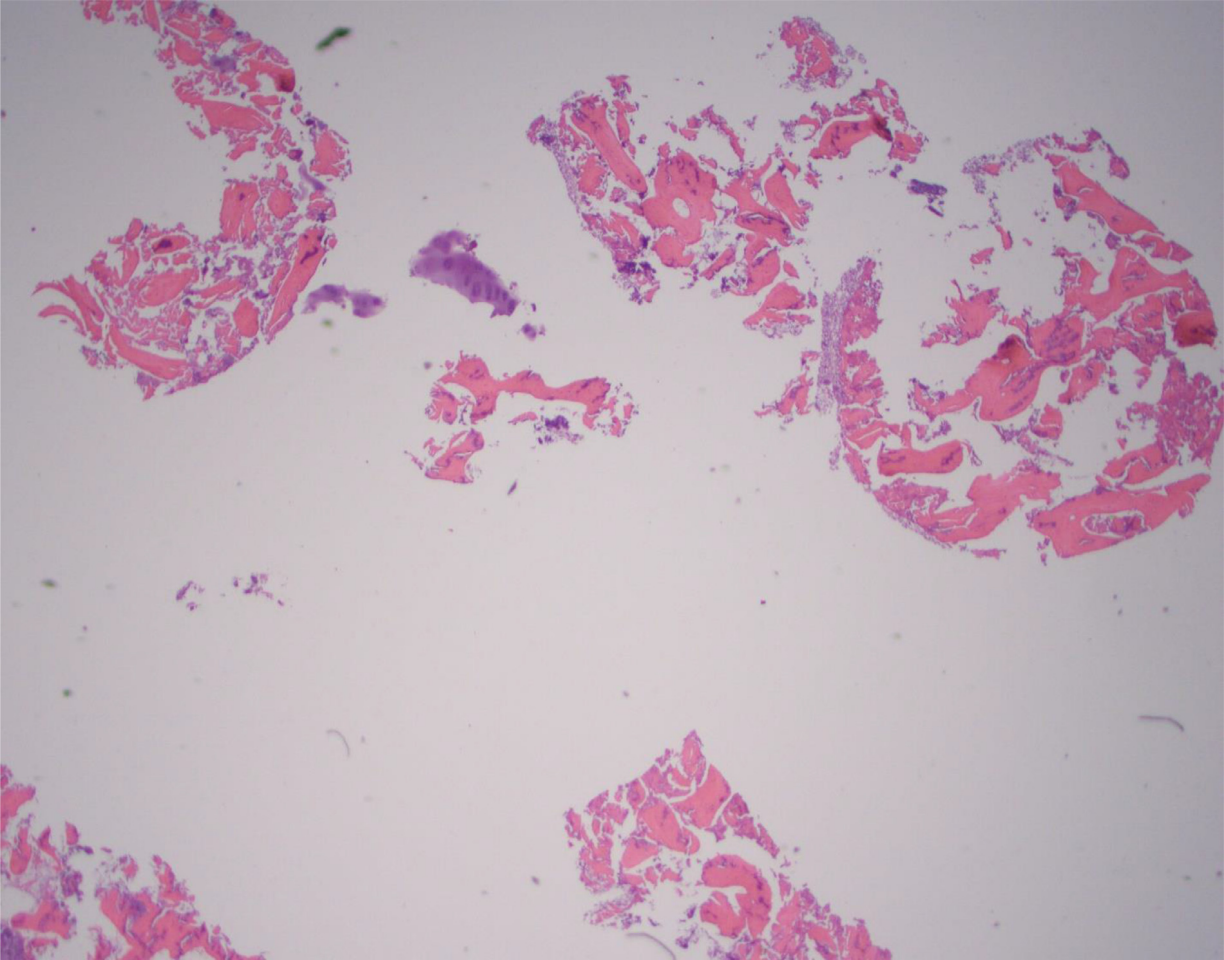
Fragmentation. Hematoxylin and eosin-stained slide at low power depicts an example of overall fragmentation of the cellular material. This was obtained with the manual technique.

All slides were reviewed by three separate pathologists (4, 10, and 12 years of experience) who were blinded to the biopsy technique used to obtain the sample. The samples were evaluated and scored for several features. First, each was analyzed for the formation of small acellular particles of bone created by the biopsy needle which may obscure the sample also known as “bone dust” (0 = absent, 1 = mild, 2 = moderate, 3 = severe). Cellular degeneration due to heat or physical injury from the biopsy was recorded as “thermal/crush artifact” (0 = absent, 1 = mild, 2 = moderate, 3 = severe). How well the individual marrow cells were visualized (cellular morphology) was graded (1 = excellent, 2= adequate, 3 = compromised). Finally, whether the core was fragmented (yes/no) and whether the sample was considered to have acceptable quality to render a diagnosis (yes or no or limited/suboptimal) were also recorded. Examples of these artifacts are shown in Figure 2, Figure 3, Figure 4, Figure 5.

### 2.3 Statistical analysis

Bayesian Multilevel Nonlinear Regression models were performed to assess the association between the three techniques across the different categories. Overall scores were averaged for each technique. Results were controlled for rater differences and nesting. The three techniques were compared to each other as follows: manual vs. pulsed powered, manual vs. full powered, and pulsed powered vs. full powered. The powered techniques (full and pulsed) were also combined into a single group and compared to the manual technique for further analysis. Using Dunn's Test of Nonparametric Comparisons, sample lengths in relation to techniques were analyzed. Inter-observer reproducibility was calculated using Cohen's weighted Kappa as well as intra-class correlation coefficients [17]. Statistics were performed using the R program (R Core Team, Vienna, Austria) [18].

## 3 Results

When comparing the manual to the pulsed powered technique, there was significantly less bone dust (p<0.0001) and better diagnostic acceptability (p=0.001) with the manual technique. There was no significant difference in thermal/crush artifact (p=0.163), cellular morphology (p=0.109), nor fragmentation (p=0.411).

When comparing the manual to the full powered technique, there was significantly less bone dust (p<0.0001), decreased fragmentation (p<0.0001), and better diagnostic acceptability (p=0.027) with the manual technique. There was no significant difference in thermal/crush artifact (p=0.421) nor cellular morphology (p=0.09).

When comparing the pulsed powered to the full powered technique, there was increased fragmentation (p<0.0001) with the pulsed powered technique. There was no significant difference in thermal/crush artifact (p=0.775), bone dust formation (p=0.594), cellular morphology (p=0.652), and diagnostic acceptability (p=0.179).

When comparing manual to the pooled powered techniques, there was significant difference across all categories with the manual technique: decreased thermal/crush artifact (p=0.014), decreased bone dust (p<0.0001), better cellular morphology (p=0.047), decreased fragmentation (p<0.0001), and better diagnostic acceptability (p<0.0001). All results are displayed in Table 1 with Tables 2 and 3 containing the individual scores from each method.

**Table 1:** Performance of Manual & Powered Techniques. Statistical results are displayed comparing techniques across the qualitative categories as labelled. Please note that pooled power includes pulsed power and full power techniques.

Performance of Manual & Powered Techniques		
*Category*	*Comparison*	*P Value*
1) Thermal/Crush Artifact	Manual vs Full Power	0.421
	Manual vs Pulsed Power	0.163
	Manual vs Pooled Power	**0.014**
	Full Power vs Pulsed Power	0.775
2) Bone Dust	Manual vs Full Power	**<0.0001**
	Manual vs Pulsed Power	**<0.0001**
	Manual vs Pooled Power	**<0.0001**
	Full Power vs Pulsed Power	0.594
3) Cellular Morphology	Manual vs Full Power	0.090
	Manual vs Pulsed Power	0.109
	Manual vs Pooled Power	**0.047**
	Full Power vs Pulsed	0.652
4) Fragmentation	Manual vs Full Power	**<0.0001**
	Manual vs Pulsed Power	0.411
	Manual vs Pooled Power	**<0.0001**
	Full Power vs Pulsed	**<0.0001**
5) Diagnostic Acceptability	Manual vs Full Power	**0.027**
	Manual vs Pulsed Power	**0.001**
	Manual vs Pooled Power	**<0.0001**
	Full Power vs Pulsed	0.179

**Table 2:** Individual scoring results. Individual scoring for each technique is displayed regarding thermal/crush artifact, bone dust, and cellular morphology.

***Category***	***Technique***	0	1	2	3
Thermal/Crush Artifact	Manual	15	5	7	3
		50.0%	16.7%	23.3%	10.0%
	Full Power	9	11	9	1
		30.0%	36.7%	30.0%	3.3%
	Pulsed Power	5	14	10	1
		16.7%	46.7%	33.3%	3.3%
Bone Dust	Manual	16	13	1	0
		53.3%	43.3%	3.3%	0.0%
	Full Power	1	6	11	12
		3.3%	20.0%	36.7%	40.0%
	Pulsed Power	1	8	14	7
		3.3%	26.7%	46.7%	23.3%
Cellular Morphology	Manual	0	20	6	4
		0.0%	66.7%	20.0%	13.3%
	Full Power	0	11	15	4
		0.0%	36.7%	50.0%	13.3%
	Pulsed Power	0	13	11	6
		0.0%	43.3%	36.7%	20.0%

**Table 3:** Additional individual scoring results. Individual scoring for each technique is displayed regarding fragmentation and diagnostic acceptability.

***Category***	***Technique***	Limited	No	Yes
Fragmentation	Manual		22	8
			73.3%	26.7%
	Full Power		13	17
			43.3%	56.7%
	Pulsed Power		0	30
			0.0%	100.0%
Diagnostic Acceptability	Manual	6	0	24
		20.0%	0.0%	80.0%
	Full Power	12	3	15
		40.0%	10.0%	50.0%
	Pulsed Power	17	5	8
		56.7%	16.7%	26.7%

The average length of the core samples was the longest for the manual technique measuring 14.60 mm. The average sample length of the full power technique was 11.50 mm with the pulsed technique cores measuring 9.10 mm. In combination the powered technique average length was 10.30 mm. There was only a significant difference in the lengths obtained between the manual technique and the pulsed technique (p=0.025).

Unweighted Kappa coefficients tested for each of the three sampling modalities indicates acceptable to good level between raters (yielding κ = 0.600, 0.477 and 0.398 for manual, pulsed powered, and full powered techniques respectively). Intra-class correlation coefficients (ICC) for each of the three sampling modalities also reflect fair to good level of rater agreement (ICC (A,1) = 0.652 [0.512, 0.770], 0.307 [0.137, 0.487], and 0.474 [0.303, 0.633]) for estimates of ICC [95% CI] using manual, pulsed power, and full power techniques respectively) [19].

## 4 Discussion

Given the results of this animal model study, there is statistically significant data showing that manual techniques produce less artifact than powered techniques and allows for a more diagnostic sample for the pathologists to analyze, in line with prior studies [7,12,20] and differing from others [5,10,13,21].

Other studies evaluated different biopsy systems [22] with a few concluding that the powered device produces longer specimens [20,23], differing from this study. Between the two systems used in this study, the manual system produced longer specimens, which was only statistically significant when comparing our manual to pulsed powered technique. This is felt to be directly related to the mechanical operation of the non-tapered powered system causing added compression of the sample during expulsion versus the tapered system of the manual system. Also, as a result, there was significantly increased fragmentation with the pulsed powered technique. The retrograde design of the manual system with the tapered biopsy tool led to decreased compaction. Perhaps obtaining more samples with smaller lengths could be beneficial in mitigating this effect when using the powered technique, as suggested by Chang et al. [24].

Analysis of the individual scores of each method reveals some important findings. In the grading of bone dust, there were no manually obtained samples reaching the highest grade. The full powered technique had twelve samples being scored the highest grade of this artifact with the pulsed powered technique having seven samples. This reinforces the significant decrease in bone dust with the manual technique. No technique received the lowest score in cellular morphology, indicating that samples were adequate across the board in this category. All of the pulsed technique samples were fragmented. Finally and importantly, all of the manual samples had some degree of diagnostic acceptability with zero samples being scored as unacceptable compared to eight samples with the powered techniques.

Results did not show any increased advantages with the pulsed technique, but perhaps a study with a throttle-controllable powered device could show differences. Typically, these devices are only available as invasive surgical tools for orthopedic surgery use.

While the manual technique produced the best specimens for pathologic analysis in this study, the powered device should not be discredited. The powered device allows for quicker access and more control working in solid bone, especially in sclerotic lesions [3,11]. If the patient's overall health is compromised, a potentially quicker procedure would be beneficial for patient safety and comfort, considering the use of sedation and often uncomfortable positioning for these procedures. In cases with significant sclerosis, one may not be able to generate enough torque or force to navigate such dense bone. A powered system would allow quick access while having no issues traversing dense bone. Once placed in the right location, the coaxially placed biopsy tool could be manually driven into the lesion to obtain samples rather than using the powered device, combining the benefits of both techniques.

Analysis of the inter-observer reproducibility resulted in acceptable/fair to good agreement. An important feature of the results is that the manual technique had higher reproducibility among all the techniques. As all raters were blinded to the study's technique and objectives, this suggests that the manual technique resulted in less variability and greater agreement, which could be attributed to the improvement of overall sample quality and consistency.

There were limitations to this study. While shown to be closely representative of human bone, lamb bone is still an animal model. Also, despite obtaining and processing specimens immediately, the tissue would still be considered post-mortem tissue. The samples were taken randomly and did not target a radiologic abnormality or lesion as would be done in a clinical setting. Only two different manufacturers were used, but these were considered good representations of the other biopsy tools available for current use. It should also be considered that bone biopsies are performed of abnormal/pathologic tissue, while it is assumed that the animal models were free from disease. The overall sample size was small.

In conclusion, manually obtained bone biopsy samples generally produce a more diagnostic sample than powered techniques in a healthy animal model. Given these results, manual bone biopsy methods should be encouraged. Despite lower quality, powered techniques still produced samples adequate for analysis and are still warranted in appropriate clinical scenarios considering lesion composition, the difficulty of access and the patient's overall condition.

## Declaration of Competing Interests

Corey K. Ho owns stock in Johnson and Johnson. M.K. Jesse serves as Medtronic faculty.

## CRediT authorship contribution statement

**Corey K Ho:** Conceptualization, Data curation, Formal analysis, Investigation, Methodology, Project administration, Resources, Supervision, Validation, Writing – original draft, Writing – review & editing. **David Gimarc:** Formal analysis, Investigation, Methodology, Resources, Validation, Writing – original draft, Writing – review & editing. **Hsieng-Feng Carroll:** Data curation, Formal analysis, Methodology, Writing – original draft, Writing – review & editing. **Michael Clay:** Data curation, Methodology, Writing – original draft, Writing – review & editing. **Jeffrey Schowinsky:** Data curation, Methodology, Writing – original draft, Writing – review & editing. **MK Jesse:** Formal analysis, Investigation, Methodology, Resources, Validation, Writing – original draft, Writing – review & editing. **Amanda M Crawford:** Conceptualization, Investigation, Methodology, Resources, Writing – original draft. **Carrie B Marshall:** Conceptualization, Data curation, Formal analysis, Investigation, Methodology, Project administration, Resources, Supervision, Validation, Writing – original draft, Writing – review & editing.
